# Solid-State Kinetic Investigations of Nonisothermal Reduction of Iron Species Supported on SBA-15

**DOI:** 10.1155/2017/6205297

**Published:** 2017-11-01

**Authors:** N. S. Genz, D. Baabe, T. Ressler

**Affiliations:** ^1^Institut für Chemie, Technische Universität Berlin, Straße des 17. Juni 135, 10623 Berlin, Germany; ^2^Institut für Anorganische und Analytische Chemie, Technische Universität Braunschweig, Hagenring 30, 38106 Braunschweig, Germany

## Abstract

Iron oxide catalysts supported on nanostructured silica SBA-15 were synthesized with various iron loadings using two different precursors. Structural characterization of the as-prepared Fe_*x*_O_*y*_/SBA-15 samples was performed by nitrogen physisorption, X-ray diffraction, DR-UV-Vis spectroscopy, and Mössbauer spectroscopy. An increasing size of the resulting iron species correlated with an increasing iron loading. Significantly smaller iron species were obtained from (Fe(III), NH_4_)-citrate precursors compared to Fe(III)-nitrate precursors. Moreover, smaller iron species resulted in a smoother surface of the support material. Temperature-programmed reduction (TPR) of the Fe_*x*_O_*y*_/SBA-15 samples with H_2_ revealed better reducibility of the samples originating from Fe(III)-nitrate precursors. Varying the iron loading led to a change in reduction mechanism. TPR traces were analyzed by model-independent Kissinger method, Ozawa, Flynn, and Wall (OFW) method, and model-dependent Coats-Redfern method. JMAK kinetic analysis afforded a one-dimensional reduction process for the Fe_*x*_O_*y*_/SBA-15 samples. The Kissinger method yielded the lowest apparent activation energy for the lowest loaded citrate sample (*E*_*a*_ ≈ 39 kJ/mol). Conversely, the lowest loaded nitrate sample possessed the highest apparent activation energy (*E*_*a*_ ≈ 88 kJ/mol). For samples obtained from Fe(III)-nitrate precursors, *E*_*a*_ decreased with increasing iron loading. Apparent activation energies from model-independent analysis methods agreed well with those from model-dependent methods. Nucleation as rate-determining step in the reduction of the iron oxide species was consistent with the Mampel solid-state reaction model.

## 1. Introduction

Metal oxide catalysts with complex chemical compositions are often employed in selective oxidation reactions [1]. Not only oxygen mobility and lattice diffusion but also redox properties of the metal oxide catalyst significantly influence performance in selective oxidation. Therefore, understanding reduction and reoxidation kinetics is a fundamental starting point for deducing reliable structure-activity correlations. Iron-containing catalysts are active in nitrogen oxides removal, Friedel-Crafts reactions, Fischer-Tropsch synthesis, catalytic methane decomposition, and selective oxidation reactions [1–7]. Moreover, redox promotors such as Fe^2+^/Fe^3+^ are used to improve redox properties of selective oxidation catalysts [1, 8, 9].

In catalysis research, more often than not, revealing reliable structure-activity correlations requires reducing chemical and structural complexity of metal oxide catalysts. Moreover, catalytic reactions occur on the surface of the catalysts, while the surface structure may differ significantly from that of the bulk. Therefore, dispersing metal oxides on well-defined support materials may result in suitable model systems. Various synthesis procedures have been used for dispersing active iron oxide species on suitable support materials. However, achieving well-dispersed and small or even isolated iron species on the support remains challenging [3]. Nanostructured silica materials, such as SBA-15, represent suitable support materials for metal oxide catalysts [10]. Furthermore, the size of the resulting species can be influenced by using various precursors for synthesis [3].

Evolution of structure and function of heterogeneous catalysts are frequently determined under nonisothermal conditions. Hence, additional solid-state kinetic analysis of experimental data measured under these conditions may be helpful in corroborating structure-activity correlations [11–14]. Experimental measurements for solid-state kinetic analysis can be performed under either isothermal or nonisothermal reaction conditions. Dependent on reaction conditions, fundamentally different analysis methods are required. Moreover, in contrast to isothermal conditions, solid-state kinetic investigations under nonisothermal conditions require a more complex mathematical analysis. In this work, we aimed at establishing solid-state kinetic analysis procedures for treating conventional temperature-programmed reduction data. Although originally intended for analyzing data measured for bulk samples, these procedures are shown to be equally useful for treating data measured for supported oxide species.

For solid-state kinetic analysis of data measured under nonisothermal conditions, two approaches can be distinguished. First, solid-state kinetic data can be analyzed by model-independent Kissinger or isoconversional method of Ozawa, Flynn, and Wall (OFW). Whereas the Kissinger method yields one apparent activation energy of the rate-determining step, the OFW method yields an evolution of apparent activation energy as function of reaction degree *α*. Model-independent kinetic analysis is not based on any model assumptions; consequently the “kinetic triple” (apparent activation energy *E*_*a*_, preexponential factor *A* of the Arrhenius-type temperature-dependence of the rate constant, and suitable solid-state reaction model *g*(*α*)) cannot be identified. Therefore, a second complementary approach to solid-state kinetic analysis is required. Model-dependent solid-state kinetic analysis employs several solid-state kinetic reaction models *g*(*α*). After identifying the suitable solid-state reaction model, the “kinetic triple” can be determined.

Here, iron oxide catalysts supported on SBA-15 as suitable model catalyst for selective oxidation were studied under various nonisothermal reaction conditions. Influence of iron loading and various precursors on structural and kinetic properties of the catalysts was investigated.

## 2. Experimental

### 2.1. Sample Preparation

Mesoporous silica SBA-15 was prepared according to Zhao et al. [10]. The surfactant, Pluronic® P123, was dissolved in a mixture of deionized water and HCl (37%) and the reaction mixture was stirred at 308 K for 24 h. Tetraethyl orthosilicate (TEOS) was added to the solution and the reaction mixture was stirred at 308 K for 24 h and then hydrothermally treated in pressure-resistant bottles at 388 K for 24 h. The obtained white solid was filtered, washed with a mixture of deionized water and ethanol (20 : 1), air-dried, and calcined. Calcination was carried out in three steps: (I) 378 K for 135 min, (II) 453 K for 3 h, and (III) 873 K for 5 h. The heating rate was kept at 1 K/min. Iron oxides supported on SBA-15 were prepared by incipient wetness technique. Therefore, an aqueous solution of (Fe(III), NH_4_)-citrate or Fe(III)-nitrate was used. After drying in air for 24 h, calcination was carried out at 723 K for 2 h. According to the iron loading and the used precursor, samples were denoted as 2.5 wt% Fe_Citrate, 6.3 wt% Fe_Citrate, 10.7 wt% Fe_Citrate, 2.0 wt% Fe_Nitrate, 7.2 wt% Fe_Nitrate, and 9.3 wt% Fe_Nitrate.

Furthermore, a mechanical mixture of SBA-15 and crystalline *α*-Fe_2_O_3_ (10.5 wt% Fe) was prepared and denoted as Fe_2_O_3_/SBA-15.

### 2.2. Nitrogen Physisorption

Nitrogen adsorption/desorption isotherms were measured at 77 K using a BELSORP-mini II (BEL Japan, Inc.). Prior to measurements, the samples were pretreated under reduced pressure (10^−2^ kPa) at 368 K for 35 min and kept under the same pressure at 448 K for 15 h (BELPREP-vac II).

### 2.3. Transmission Electron Microscopy

Transmission electron microscopy (TEM) images were recorded on a FEI Tecnai G^2^ 20 S-TWIN microscope equipped with a LaB_6_ cathode and a 1k × 1k CCD camera (GATAN MS794). Acceleration voltage was set to 220 kV and samples were prepared on 300 mesh Cu grids with Holey carbon film.

### 2.4. Powder X-Ray Diffraction

Powder X-ray diffraction patterns were obtained using an X'Pert PRO diffractometer (PANalytical, 40 kV, 40 mA) in theta/theta geometry equipped with a solid-state multichannel detector (PIXel). Cu K*α* radiation was used. Wide-angle diffraction scans were collected in reflection mode. Small-angle diffraction patterns were measured in transmission mode between 0.4° and 6° 2*θ*in steps of 0.013° 2*θ* with a sampling time of 90 s/step.

### 2.5. Diffuse Reflectance UV-Vis Spectroscopy

Diffuse reflectance UV-Vis (DR-UV-Vis) spectroscopy was conducted on a two-beam spectrometer (V-670, Jasco) using a barium sulfate coated integration sphere (scan speed: 100 nm/min; slit width: 5.0 nm (UV-Vis) and 20 nm (NIR); and spectral region: 2000–220 nm). SBA-15 was used as white standard for all samples.

### 2.6. Mössbauer Spectroscopy

Zero-field ^57^Fe Mössbauer spectroscopic measurements were conducted on a transmission spectrometer with sinusoidal velocity sweep. Velocity calibration was done with an *α*-Fe foil at ambient temperature. Measurements of samples 2.0 wt% Fe_Nitrate and 7.2 wt% Fe_Nitrate were performed using a Janis closed-cycle cryostat with the sample container entirely immersed in Helium exchange gas at 14 and 300 K. Combined with measurements over a time period of about one to twelve days, the helium exchange gas ensured a gradient-free sample temperature. The sample temperature was recorded with a calibrated Si diode located close to the sample container made of Teflon or PEEK (polyether ether ketone), providing a temperature stability of better than 0.1 K. Additional measurements of samples 9.3 wt% Fe_Nitrate, 7.2 wt% Fe_Nitrate, 2.0 wt% Fe_Nitrate, and 10.7 wt% Fe_Citrate were carried out on a spectrometer equipped with a Cryovac continuous flow cryostat with comparable specifications, geometry, and sample environment as described above. The nominal activity of the Mössbauer sources used was about 50 mCi of ^57^Co in a rhodium matrix. Spectra at 4 K were recorded every 30 minutes during overall measurement duration. Each Mössbauer spectrum shown here corresponds to the last spectrum in the respective series. Quantitative analysis of the recorded spectra was conducted on basis of the stochastic relaxation model developed by Blume and Tjon [15], in which the magnetic hyperfine field *B*_hf_ fluctuates randomly between two directions (+*B*_hf_ and −*B*_hf_) along the symmetry axis of an axially symmetric electric field gradient tensor. Using this model is motivated by the observation of a significant line broadening, in particular in the spectra obtained for 7.2 wt% Fe_Nitrate at intermediate temperatures of ca. 60 and 100 K, suggesting the presence of slow relaxation processes with relaxation times*τ*_*c*_ that are long or of the same order of magnitude as the Larmor precession time of the ^57^Fe nuclear magnetic moment (i.e., 10^−6^ s <*τ*_*c*_ < 10^−8^ s). The quadrupole shift *ε* is given by *e*^2^*qQ*/4, assuming that *e*^2^*qQ* ≪ *μB*_hf_ (constants *μ*, *e*, *q*, and *Q* were used in their usual meaning). The isomer shift*δ* is reported with respect to iron metal at ambient temperature and was not corrected in terms of the second-order Doppler shift.

### 2.7. Temperature-Programmed Reduction

Temperature-programmed reduction (TPR) was performed using a BELCAT-B (BEL Japan, Inc.). Samples were placed on silica wool in a silica glass tube reactor. Evolving water was trapped using a molecular sieve (4 Å). Gas mixture consisted of 5% H_2_ in 95% Ar with a total gas flow of 40 ml/min. Heating rates used were 5, 10, 15, and 20 K/min to 1223 K. A constant initial sample weight was used and H_2_ consumption was continuously monitored by a thermal conductivity detector.

## 3. Results and Discussion

### 3.1. Sample Characterization

#### 3.1.1. Nitrogen Physisorption Measurements

**Figure 1:**
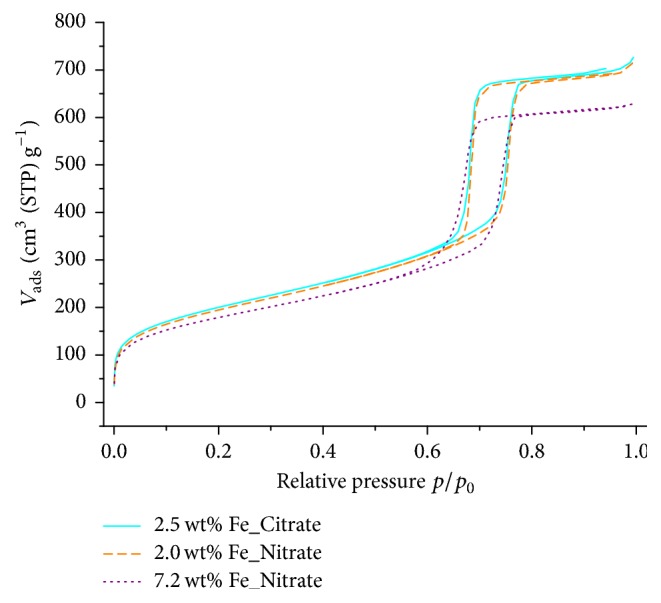
Nitrogen adsorption/desorption isotherms of the samples 2.5 wt% Fe_Citrate (straight lines), 2.0 wt% Fe_Nitrate (dashed lines), and 7.2 wt% Fe_Nitrate (dotted lines).

**Figure 2:**
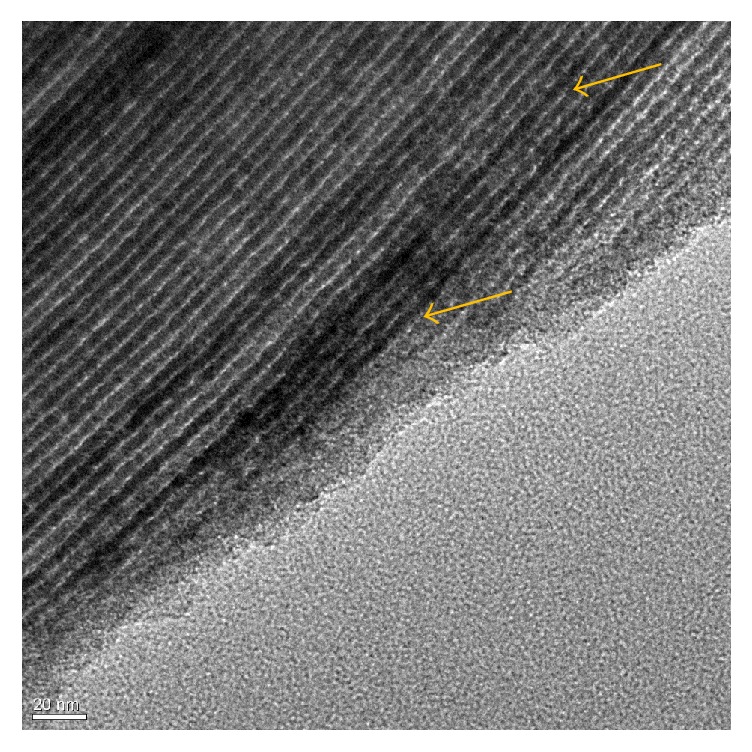
TEM micrograph of sample 9.3 wt% Fe_Nitrate. Dark contrast (arrows) indicates the iron species.

Fe_*x*_O_*y*_/SBA-15 samples and support material SBA-15 exhibited type IV nitrogen adsorption/desorption isotherms indicating mesoporous materials (Figure 1). Adsorption and desorption branches were nearly parallel at the hysteresis loop, as expected for regularly shaped pores. Both SBA-15 and Fe_*x*_O_*y*_/SBA-15 samples exhibited high specific surface areas with narrow pore size distributions. Independent of the used precursor, low loaded Fe_*x*_O_*y*_/SBA-15 samples showed significantly higher specific surface areas than higher loaded samples. Compared to SBA-15, all Fe_*x*_O_*y*_/SBA-15 samples showed a decrease in specific surface area. Whereas SBA-15 possessed a BET-surface between 743.4 and 779.4 m^2^/g, those of the Fe_*x*_O_*y*_/SBA-15 samples were determined to be between 605.9 and 725.2 m^2^/g. Pore size distribution was calculated by the BJH method and revealed a decrease in pore radius from 4.6 nm of SBA-15 to 4.0 nm of Fe_*x*_O_*y*_/SBA-15 samples. This decrease in specific surface area as well as the decrease in pore radius with increasing iron loading indicated the presence of iron species in the mesopores of SBA-15. Moreover, transmission electron microscopy (TEM) measurements of the Fe_*x*_O_*y*_/SBA-15 samples also indicated that iron species were located in the pore system of SBA-15 with no iron species detected on the external surface of SBA-15. TEM micrograph of the highest loaded nitrate sample, 9.3 wt% Fe_Nitrate, is depicted in Figure 2. The dark contrast (arrows in Figure 2) indicated the presence of iron species in the pore channels of SBA-15.

In addition to BET method and BJH method, the modified FHH method was used to analyze the nitrogen physisorption data. Herein, the fractal dimension *D*_*f*_ was determined as a measure of the roughness of the surface [16, 17]. For Fe_*x*_O_*y*_/SBA-15 samples, as well as SBA-15, the fractal dimension was between 2 and 3. This indicated a rough surface. In order to elucidate the effect of supported iron species on surface roughness of the support material, Δ*D*_*f*_ values were calculated as difference from *D*_*f*_ values of SBA-15 and those of the corresponding Fe_*x*_O_*y*_/SBA-15 samples. In contrast to the nitrate samples, citrate samples possessed significantly higher values of Δ*D*_*f*_ (Tables 1 and 2). Therefore, compared to those of the nitrate samples, the surface of the citrate samples appeared to be smoother. A possible explanation for the differences in surface roughness of the support material might be the differently pronounced chelating effect of the two precursors. The citrate precursor showed a more pronounced chelating effect and, therefore, stronger bonds between citrate ligands and Fe(III) central atoms. Due to the stronger bonds between citrate ligands and Fe(III) atoms, polydentate citrate ligands encapsulated the Fe(III) ions, thereby preventing agglomeration of iron species during calcination. Thus, after calcination and removal of the citrate ligands, the resulting Fe(III) species were more isolated and dispersed on the support material. Conversely, nitrate ligands showed minor interactions with the Fe(III) ions due to the less pronounced chelating effect. Therefore, nitrate removal during calcination was facilitated and the resulting Fe(III) species readily aggregated and formed less dispersed iron oxide species on the support material [16, 17].

#### 3.1.2. X-Ray Diffraction

**Figure 3:**
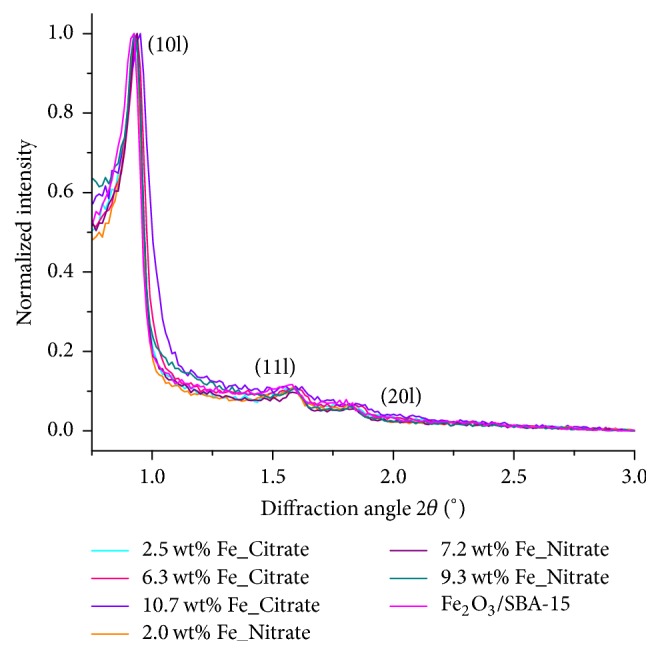
Small-angle X-ray diffraction patterns of all Fe_*x*_O_*y*_/SBA-15 samples and the mechanical mixture Fe_2_O_3_/SBA-15.

**Figure 4:**
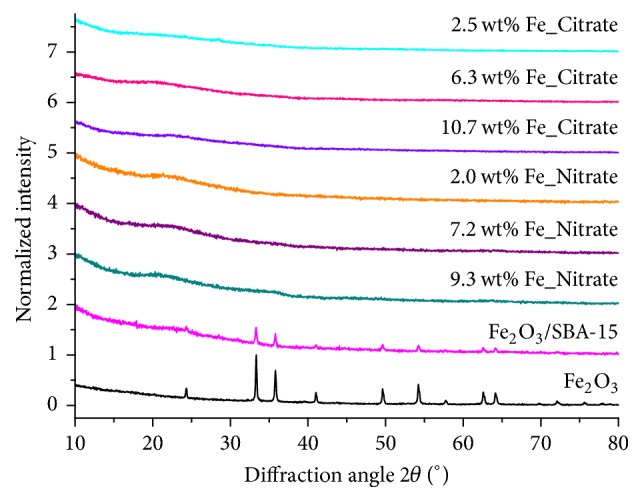
Wide-angle X-ray diffraction patterns of all Fe_*x*_O_*y*_/SBA-15 samples, a reference (mechanically mixed SBA-15 and crystalline Fe_2_O_3_), and crystalline Fe_2_O_3_.

Figure 3 depicts the small-angle XRD patterns of the Fe_*x*_O_*y*_/SBA-15 samples and the mechanical mixture Fe_2_O_3_/SBA-15. Diffraction peaks (10l), (11l), and (20l) correspond to the two-dimensional hexagonal symmetry of SBA-15. The diffraction peaks were visible for all samples and the mechanical mixture Fe_2_O_3_/SBA-15. Wide-angle X-ray diffraction patterns of the Fe_*x*_O_*y*_/SBA-15 samples showed no long-range ordered phases indicative of small and isolated iron species (Figure 4). Conversely, XRD patterns of the mechanical mixture of SBA-15 and Fe_2_O_3_ showed diffraction peaks of crystalline Fe_2_O_3_.

#### 3.1.3. Diffuse Reflectance UV-Vis Spectroscopy

**Figure 5:**
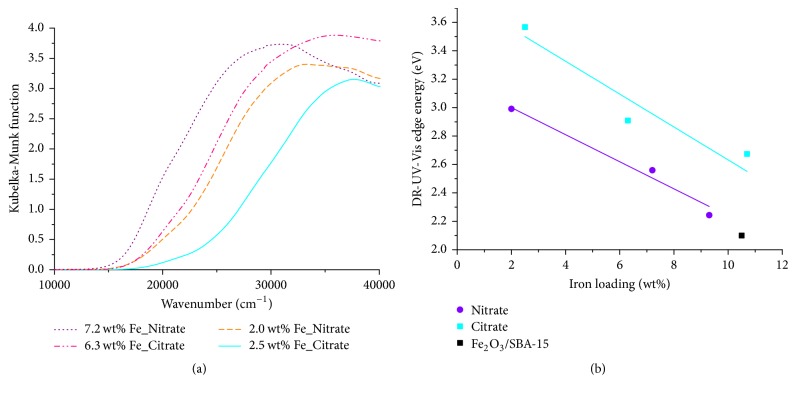
(a) DR-UV-Vis spectra of 2.5 wt% Fe_Citrate (straight line), 2.0 wt% Fe_Nitrate (dashed line), 6.3 wt% Fe_Citrate (dashed double-dotted line), and 7.2 wt% Fe_Nitrate (dotted line). (b) Edge energy as function of iron loading for nitrate samples (circles), citrate samples (squares), and mechanical mixture (square).

DR-UV-Vis spectra of the Fe_*x*_O_*y*_/SBA-15 samples are depicted in Figure 5(a). Independent of the utilized precursor, a red-shift and broadening of the absorption bands with increasing iron loading can be seen (Figure 5(a)). The red-shift of the absorption and, thus, a decreasing edge energy with increasing iron loading can be correlated with an aggregation of Fe(III) species [18, 19]. All Fe_*x*_O_*y*_/SBA-15 samples possessed edge energy values higher than 2.1 eV (edge energy in the DR-UV-Vis spectrum of crystalline Fe_2_O_3_). Hence, the size of the supported iron species was smaller than that of crystalline Fe_2_O_3_ in all samples. Both citrate samples and nitrate samples exhibited a decrease in edge energy with increased iron loading (Figure 5(b)). However, the citrate samples showed higher edge energy values than the corresponding nitrate samples. Therefore, iron species obtained by citrate precursor were smaller compared to those obtained by nitrate precursor.

#### 3.1.4. Mössbauer Spectroscopy

**Figure 6:**
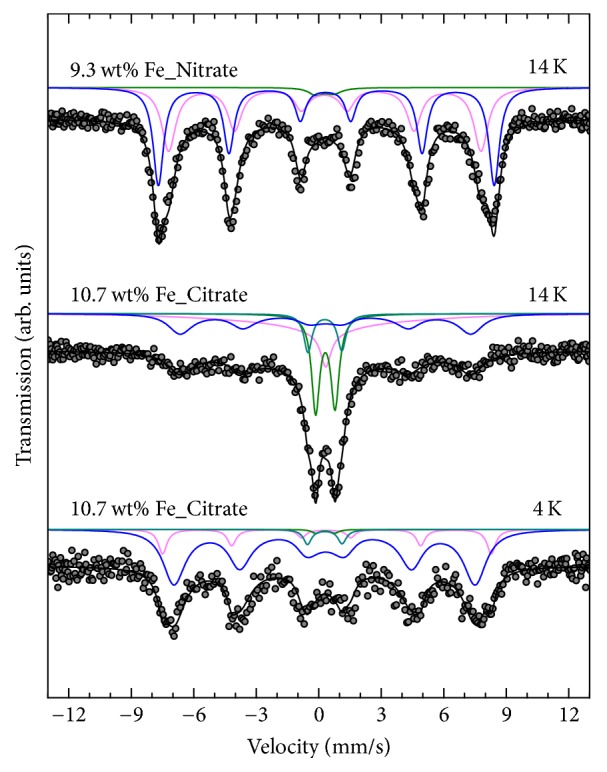
Mössbauer spectra of 9.3 wt% Fe_Nitrate (top) and 10.7 wt% Fe_Citrate (middle and bottom) at 14 and 4 K. Dots: experimental data; lines: fit curves based on stochastic Blume-Tjon relaxation model.

Mössbauer spectra of 9.3 wt% Fe_Nitrate and 10.7 wt% Fe_Citrate recorded above 200 K showed a broadened and asymmetric doublet independent of the used precursor. Therefore, this doublet was analyzed using two nonequivalent Fe sites. The determined values for the isomer shift*δ* and the quadrupole shift*ε* are consistent with those reported for superparamagnetic particles of Fe_2_O_3_ [20, 21]. At low temperatures, that is, at 14 K for 9.3 wt% Fe_Nitrate and at 4 K for 10.7 wt% Fe_Citrate (Figure 6), the doublet almost disappeared and a magnetically split hyperfine pattern was detected. This observation indicated the presence of small superparamagnetic iron oxidic species. The related Mössbauer parameters (Table 3) are furthermore consistent with those reported for (magnetically blocked) superparamagnetic particles with a local geometry similar to Fe_2_O_3_ supported on SBA-15 [20]. Therefore, a blocking temperature lower than 200 K implied an upper limit for the Fe species diameter of <10 nm [22] for 9.3 wt% Fe_Nitrate. Conversely, the observation of an almost complete blocking at lower temperatures for 10.7 wt% Fe_Citrate compared to 9.3 wt% Fe_Nitrate suggested a significantly smaller species size obtained from citrate precursor. Furthermore, refinement of the Mössbauer spectra of 9.3 wt% Fe_Nitrate and 7.2 wt% Fe_Nitrate at 14 K and of 10.7 wt% Fe_Citrate at 14 K and 4 K required an additional component (Table 3), indicating a bimodal particle size distribution.

**Figure 7:**
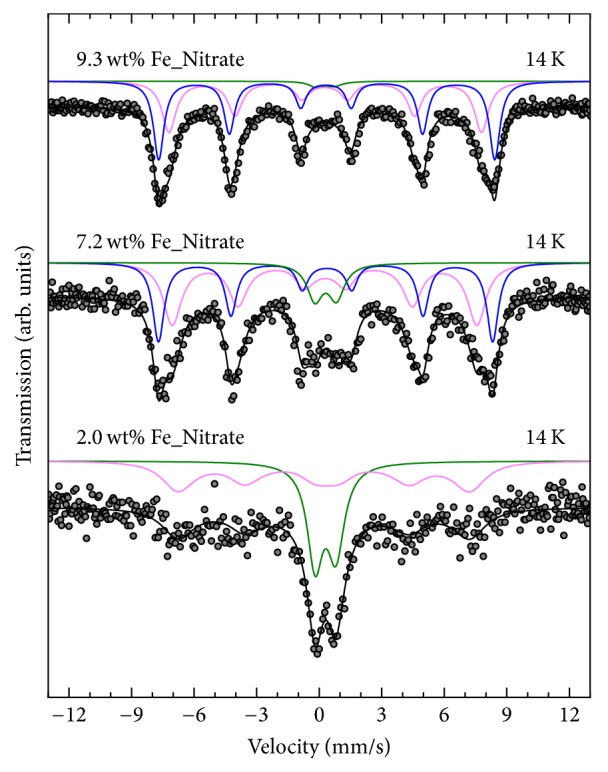
Mössbauer spectra of 9.3 wt% Fe_Nitrate (top), 7.2 wt% Fe_Nitrate (middle), and 2.0 wt% Fe_Nitrate (bottom) at 14 K. Dots: experimental data; lines: fit curves based on stochastic Blume-Tjon relaxation model.

The Mössbauer spectra of the lower loaded nitrate samples, 7.2 wt% Fe_Nitrate and 2.0 wt% Fe_Nitrate, also exhibited a broadened and asymmetric doublet at 300 K with similar values for isomer shift and quadrupole splitting as determined for 9.3 wt% Fe_Nitrate. While this doublet almost completely transformed into a magnetically split sextet for sample 9.3 wt% Fe_Nitrate at 14 K (vide supra), this transformation remained incomplete in the Mössbauer spectra of 7.2 wt% Fe_Nitrate and 2.0 wt% Fe_Nitrate at 14 K (Figure 7). The determined site population ratio of the doublet relative to the magnetically split sextet signal increased systematically with decreasing iron loading from about 2 : 98 for 9.3 wt% Fe_Nitrate to 45 : 55 for 2.0 wt% Fe_Nitrate (Table 3). Furthermore, a similar trend was observed for the determined values of the local magnetic hyperfine field (i.e., decreasing *B*_hf_ with decreasing Fe loading). Assuming that all iron in the nitrate samples consisted of iron oxide, both results independently suggested a correlation of increasing average iron species size and increasing iron loading within the nitrate samples.

#### 3.1.5. Temperature-Programmed Reduction

**Figure 8:**
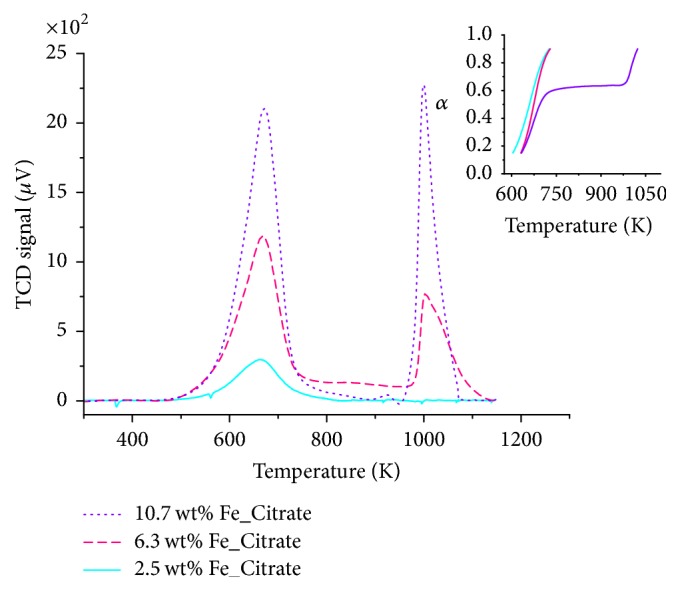
TPR traces of 2.5 wt% Fe_Citrate (straight line), 6.3 wt% Fe_Citrate (dashed line), and 10.7 wt% Fe_Citrate (dotted line) measured in 5% H_2_ in 95% argon at 10 K/min. Inset depicts reduction degree traces with increasing iron loading from left to right.

**Figure 9:**
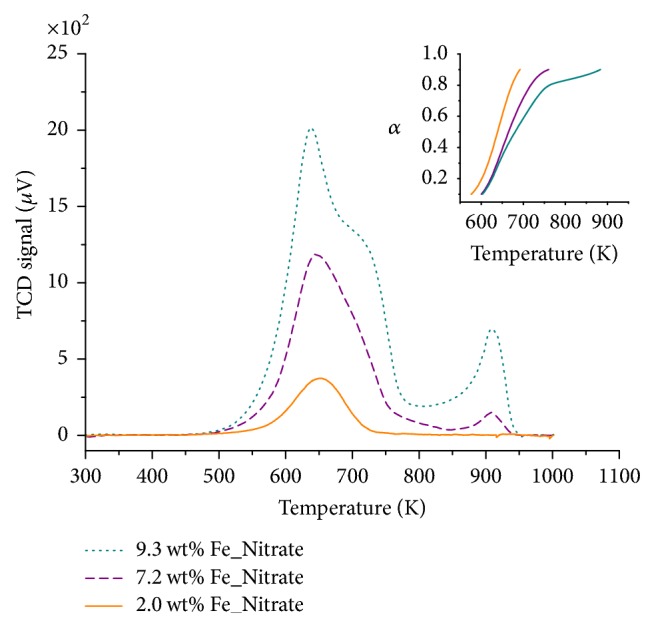
TPR traces of 2.0 wt% Fe_Nitrate (straight line), 7.2 wt% Fe_Nitrate (dashed line), and 9.3 wt% Fe_Nitrate (dotted line) measured in 5% H_2_ in 95% argon at 10 K/min. Inset depicts reduction degree traces with increasing iron loading from left to right.

Figures 8 and 9 depict TPR traces of Fe_*x*_O_*y*_/SBA-15 samples measured during reduction with H_2_ at a heating rate of 10 K/min. Significant differences in reduction profiles are discernible. Lowest loaded citrate and nitrate samples possessed one single reduction peak. Conversely, higher loaded citrate samples showed a two-step reduction (not considering a very small second TPR peak for sample 10.7 wt% Fe_Citrate), while higher loaded nitrate samples showed a three-step reduction. The first reduction step can be assigned to the reduction of Fe(III) oxidic species to Fe(II) oxidic species. The small iron species of the lowest loaded citrate and nitrate sample interacted strongly with the surface of SBA-15, preventing further reduction in the applied temperature range. Hence, these samples showed only one single reduction peak in the TPR profile. Conversely, the larger iron species in the higher loaded citrate and nitrate samples exhibited further reduction of the Fe(II) species and, hence, a two-step or even three-step reduction mechanism. Thus, increasing iron loading resulted in weaker interactions between iron species and support material.

**Figure 10:**
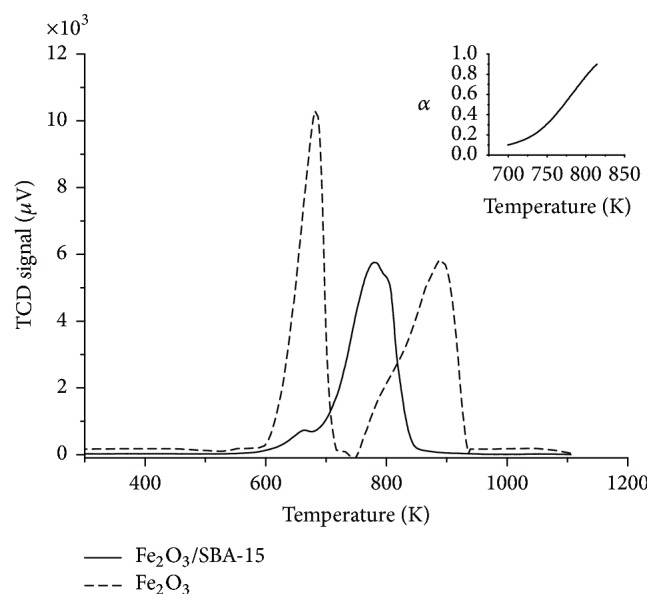
TPR traces of mechanical mixture Fe_2_O_3_/SBA-15 (straight line) and crystalline Fe_2_O_3_ (dashed line) measured in 5% H_2_ in 95% argon at 10 K/min. Inset depicts reduction degree trace.

For both, nitrate and citrate samples, an increasing temperature of the first TPR maxima correlated with an increasing iron loading. Furthermore, nitrate samples showed a shift of the TPR maxima to lower temperatures compared to the citrate samples. This shift of the TPR maxima indicated better reducibility of the nitrate samples. The mechanical mixture Fe_2_O_3_/SBA-15 exhibited two TPR maxima with a shoulder at the second TPR peak, indicating a three-step reduction (Figure 10). TPR traces of the mechanical mixture differed significantly from those of the Fe_*x*_O_*y*_/SBA-15 samples. Moreover, neither the Fe_*x*_O_*y*_/SBA-15 samples nor the mechanical mixture showed a TPR profile characteristic for crystalline Fe_2_O_3_ (Figure 10). Differences in the TPR profiles of the mechanical mixture and crystalline Fe_2_O_3_ resulted from differences in both particle sizes and dispersion of Fe_2_O_3_ crystallites [23]. Dispersion of smaller Fe_2_O_3_ crystallites on SBA-15 in the mechanical mixture compared to pure Fe_2_O_3_ induced a decreased first TPR peak and a shift of the second TPR peak to lower temperature. Significantly smaller Fe_2_O_3_ crystallites of the mechanical mixture correlated with a significantly decreased first reduction peak [23].

### 3.2. Reduction Kinetics under Nonisothermal Conditions

In the following, a more detailed solid-state kinetic analysis of the reduction traces is presented. Besides TPR traces of all nitrate samples, those of the mechanical mixture and the lowest loaded citrate sample were analyzed. After transforming TPR traces to reduction degree *α* traces, model-independent and model-dependent solid-state kinetic analysis methods were applied.

All Fe_*x*_O_*y*_/SBA-15 samples showed symmetrically shaped TPR profiles. This indicates no rate limitation by removal of the small amount of H_2_O formed by reduction of the low concentration of iron species on SBA-15. Additionally, mass transport limited processes exhibit characteristic apparent activation energies of less than 10 kJ/mol [24]. Apparent activation energies for all Fe_*x*_O_*y*_/SBA-15 samples were significantly higher than 10 kJ/mol. Therefore, mass transport limitation of reactant gas H_2_ was considered to be not rate-limiting in the reduction of Fe_*x*_O_*y*_/SBA-15.

#### 3.2.1. Kissinger Method

**Figure 11:**
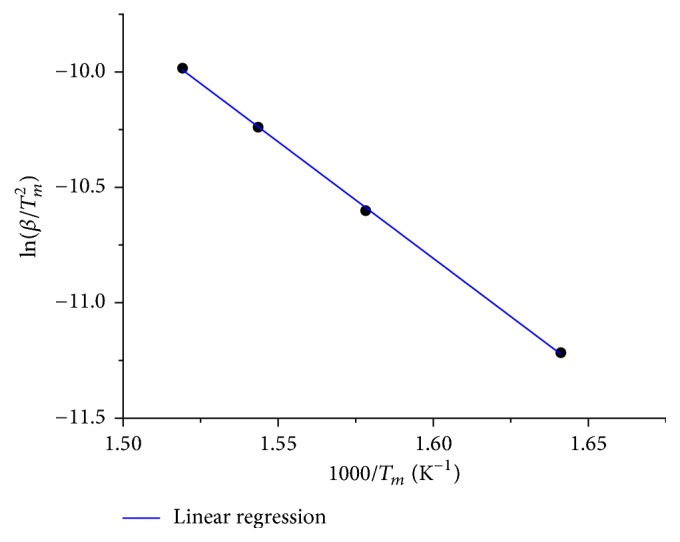
Kissinger plot for 7.2 wt% Fe_Nitrate sample extracted from TPR traces measured during reduction (5% H_2_ in 95% argon).

Apparent activation energy *E*_*a*_ of the rate-determining step during reduction was determined by applying the Kissinger method. Therefore, ln⁡[*β*/*T*_*m*_^2^] was depicted as function of 1/*T*_*m*_ [11, 25]. Here, *T*_*m*_ corresponded to the first maximum of the TPR traces (Figures 8–10). From the slope of the resulting straight line, the apparent activation energy for the reduction of Fe_*x*_O_*y*_/SBA-15 was calculated (Figure 11). The lowest loaded citrate sample possessed the lowest apparent activation energy of 39 ± 8 kJ/mol. The highest apparent activation energy of 88 ± 8 kJ/mol was calculated for sample 2.0 wt% Fe_Nitrate (Table 4). Increasing the iron loading of the nitrate samples resulted in a decreasing apparent activation energy of the rate-determining step during reduction. Moreover, results of the Kissinger method also correlated with the species size resulting from DR-UV-Vis and Mössbauer spectroscopy. Hence, increasing size of the iron species of the nitrate samples was accompanied by better reducibility and a decreasing apparent activation energy of reduction. The apparent activation energy of the mechanical mixture was calculated to be 59 ± 7 kJ/mol. This lower apparent activation energy compared to the nitrate samples was consistent with a further increased species size.

#### 3.2.2. Method of Ozawa, Flynn, and Wall

**Figure 12:**
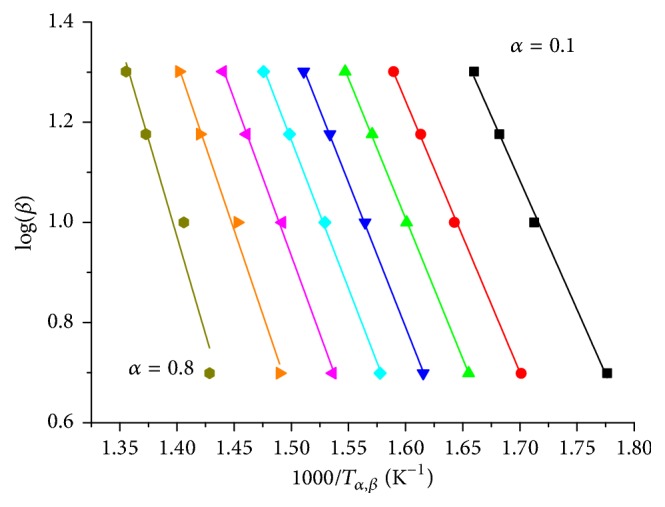
Logarithmic heating rate*β* as function of reciprocal temperature for the reduction of 7.2 wt% Fe_Nitrate in 5% H_2_ in 95% argon and reduction degree range from 0.1 to 0.8 (OFW method).

**Figure 13:**
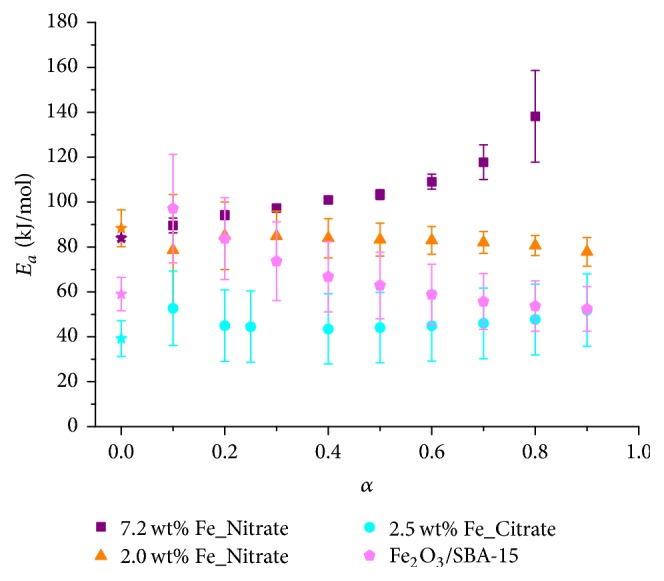
Apparent activation energy *E*_*a*_ as function of reduction degree *α* for the reduction of 2.5 wt% Fe_Citrate (circles), 2.0 wt% Fe_Nitrate (triangles), 7.2 wt% Fe_Nitrate (squares), and Fe_2_O_3_/SBA-15 (pentagons) in 5% H_2_ in 95% argon (with Senum-Yang approximation). Apparent activation energies as determined from Kissinger method are indicated at *α* = 0 (stars).

A single apparent activation energy value resulting from the Kissinger method may not be sufficient for a detailed kinetic analysis of a solid-state reaction. Therefore, the isoconversional, model-independent OFW method was applied for determining the evolution of the apparent activation energy of the rate-determining step as function of reduction degree *α* [11, 26–28]. Reduction degree *α* traces were extracted by integration of the TPR traces measured at various heating rates *β*. First, temperatures *T*_*α*,*β*_ for defined reduction degrees *α* were determined from the experimental*α* traces at various heating rates. Temperatures *T*_*α*,*β*_ were determined for reduction degrees in the range of 0.1 and 0.8, with Δ*α* = 0.1. Second, decade logarithm of the heating rate as function of 1000/*T*_*α*,*β*_ for the different reduction degrees was calculated based on

(1) $$\begin{array}{c} \log\left(\;\beta \;\right)=\log\left(\;\frac{A_{\alpha }E_{a,\alpha }}{g\left(\alpha \right)R}\;\right)-2.315-0.457\frac{E_{a,\alpha }}{RT_{\alpha ,\beta }}, \end{array}$$

with heating rate *β*, preexponential (frequency) factor *A*_*α*_ at reduction degrees *α*, apparent activation energy at reduction degrees *α*  *E*_*a*,*α*_, integral solid-state reaction model *g*(*α*), gas constant *R*, and temperatures *T*_*α*,*β*_.  Figure 12 shows the resulting straight lines for heating rates of 5, 10, 15, and 20 K/min and various reduction degrees *α*. Linear regression of the resulting straight lines resulted in apparent activation energy as a function of reduction degree *α*. Because of *E*_*a*,*α*_/*RT*_*α*,*β*_ < 20, the apparent activation energy was corrected according to Senum-Yang [11, 26]. The resulting apparent activation energy together with the apparent activation energy determined by Kissinger method is depicted in Figure 13.

The apparent activation energy obtained from the Kissinger method for samples 2.5 wt% Fe_Citrate and 2.0 wt% Fe_Nitrate agreed with the apparent activation energy obtained from the OFW method (Figure 13). Furthermore, apparent activation energies *E*_*a*_(*α*) of the lowest loaded citrate and nitrate samples were invariant in the *α* range within the error limits. Thus, a single-step reduction mechanism was assumed for the lowest loaded Fe_*x*_O_*y*_/SBA-15 samples corresponding to the single reduction peak in the TPR profiles of these samples (Figures 8 and 9). Such a reaction mechanism is more similar to homogeneous kinetics than to complex heterogeneous kinetics. Compared to the lowest loaded citrate and nitrate samples, 7.2 wt% Fe_Nitrate differed not only in the higher apparent activation energy values but also in the evolution of the apparent activation energy as function of reduction degree. The increase of the apparent activation energy may indicate a change in rate-determining step during a more complex reduction mechanism [29]. Moreover, such a more complex reduction mechanism correlated with the multistep TPR profile due to the presence of larger, weakly interacting iron species for sample 7.2 wt% Fe_Nitrate (Figure 9).

#### 3.2.3. Coats-Redfern Method

In addition to the model-independent Kissinger and OFW methods, the model-dependent Coats-Redfern [30] method provided a complementary analysis of nonisothermal kinetic data. Compared to a model-independent kinetic analysis, model-dependent analysis enables a more detailed characterization of the reaction mechanism. Here, resulting activation energies are based on assuming a suitable solid-state kinetic model. The Coats-Redfern method can be expressed by

(2) $$\begin{array}{c} \ln\left(\;\frac{g\left(\alpha \right)}{T^{2}}\;\right)=\ln\left(\;\frac{AR}{\beta E_{a}}\left[\;1-\left(\;\frac{2RT}{E_{a}}\;\right)\;\right]\;\right)-\frac{E_{a}}{RT}, \end{array}$$

with the integral solid-state reaction model *g*(*α*), temperature *T*, heating rate *β*, apparent activation energy of rate-determining step *E*_*a*_, gas constant *R*, and preexponential (frequency) factor* A*. Plotting ln⁡[*g*(*α*)/*T*^2^] as function of reciprocal temperature results in straight lines for suitable solid-state reaction models. Linear regression was conducted to determine the apparent activation energy. Here, only reaction models *g*(*α*) resulting in both suitable apparent activation energies and good linear regressions were selected for further analysis [30, 31].

For the reduction of 2.5 wt% Fe_Citrate, 2.0 wt% Fe_Nitrate, 7.2 wt% Fe_Nitrate, and the mechanical mixture Fe_2_O_3_/SBA-15, reduction degree *α* curves were analyzed. Applied solid-state reaction models were nucleation models, including power law models (P) and Avrami-Erofeyev models (A), as well as the autocatalytic Prout-Tompkins model (B1). Furthermore, diffusion models (D), geometrical contraction models (R), and reaction order-based models (F) were tested [31]. D4, F1, A2, R2, and B1 solid-state reaction models revealed wide linear ranges by plotting ln⁡[*g*(*α*)/*T*^2^] as function of reciprocal temperature for sample 2.5 wt% Fe_Citrate. Apparent activation energies for those models as obtained from the slope of the resulting straight lines are given in Table 5.

Compared to the results of the Kissinger and OFW methods, apparent activation energies at different heating rates were significantly higher for the D4 model and significantly lower for the A2 model. Hence, D4 and A2 reaction models were not considered for further analysis. The B1 model (i.e., Prout-Tompkins model) yielded apparent activation energies similar to those obtained from Kissinger and OFW methods. However, the autocatalysis B1 model assumes that defects formed at the reaction interface during nuclei growth further catalyze and, hence, accelerate the reaction. This concept appears hardly applicable to Fe_*x*_O_*y*_/SBA-15 samples with dispersed Fe species located in a nanostructured pore system. Therefore, the B1 model was also not further considered. Similar constraints hold for the R2 model. The R2 reaction model is described as geometrical contracting model in which nucleation occurs on the surface of the cylindrical crystal. Thus, the reaction rate is determined by the decreasing interface area between reactant and product phase during reaction [31]. Again, such a concept seems not applicable for small and dispersed iron species on the surface of porous support. Consequently, the F1 model was chosen as suitable reaction model for the lowest loaded citrate and nitrate samples, as well as for sample 7.2 wt% Fe_Nitrate.

The first-order reaction model (F1, Mampel model) describes solid-state reactions with a large number of nucleation sites resulting in fast nucleation. Apparently, reduction of Fe_*x*_O_*y*_/SBA-15 samples was inhibited neither by limited mobility of reactants nor by increasing product layer. Order-based reaction models are the simplest solid-state reaction models similar to those used in homogeneous kinetics where ions in solution interact weakly with each other [31, 32]. Because the Fe(III) species of the Fe_*x*_O_*y*_/SBA-15 samples constituted small and isolated nucleation sites, the F1 model can be readily applied to these samples.

For the mechanical mixture Fe_2_O_3_/SBA-15, an R3 model was a suitable reaction model. The R3 model is denoted as contracting volume model with nucleation occurring rapidly on the surface of the particles. This reaction model was consistent with a mixture of Fe_2_O_3_ crystallites and SBA-15 material as obtained by conventional sample characterization.

#### 3.2.4. JMAK Kinetics

**Figure 14:**
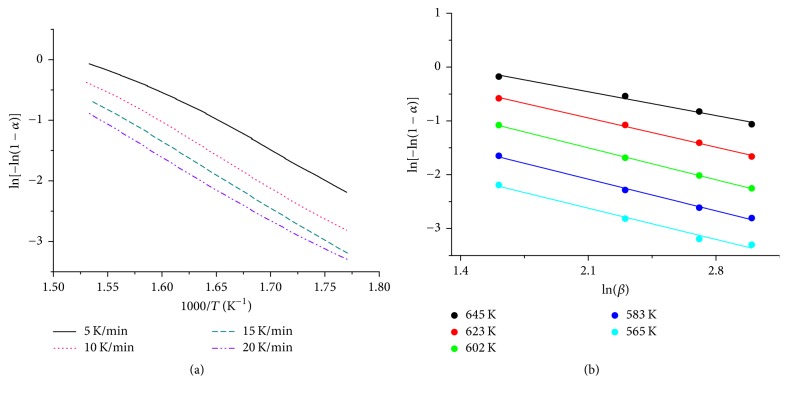
(a) ln[−ln(1 − *α*)] as function of 1000/*T* according to JMAK kinetics for determining the topological dimension of the reduction of 7.2 wt% Fe_Nitrate (5% H_2_ in 95% argon). (b) ln[−ln(1 − *α*)] as function of ln⁡(*β*) according to JMAK kinetics in order to determine the Avrami exponent for sample 7.2 wt% Fe_Nitrate.

**Figure 15:**
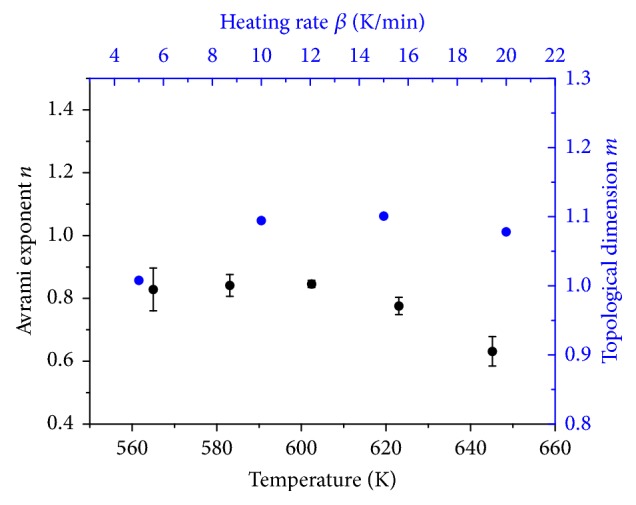
Topological dimension and Avrami exponent from JMAK kinetic analysis as function of temperature and heating rate for sample 7.2 wt% Fe_Nitrate.

**Figure 16:**
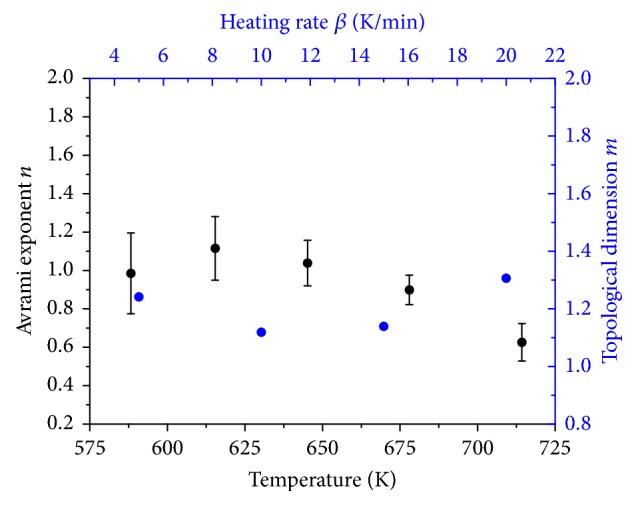
Topological dimension and Avrami exponent from JMAK kinetic analysis as function of temperature and heating rate for sample 2.0 wt% Fe_Nitrate.

In order to enable a geometrical description of the reduction reaction under nonisothermal conditions, Johnson-Mehl-Avrami-Kolmogorov (JMAK) kinetic analysis was applied [33, 34]. JMAK kinetics are based on the following equation:

(3) $$\begin{array}{c} \ln\left[\;-\ln\left(\;1-\alpha \;\right)\;\right]=-n\ln\left(\;\beta \;\right)-1.052\frac{mE}{RT}+\text{Const.}, \end{array}$$

with heating rate *β*, apparent activation energy of the rate-determining step *E*, temperature *T*, gas constant *R*, reduction degree *α*, topological dimension* m,* and Avrami exponent* n*. Plotting ln⁡[−ln⁡(1 − *α*)] as function of reciprocal temperature at different heating rates resulted in straight lines (Figure 14(a)). From the slope of the resulting straight lines, the topological dimension *m* can be determined. Here, the apparent activation energy obtained by the Kissinger method was inserted in (3). Based on (3), the Avrami exponent *n* is derived according to

(4) $$\begin{array}{c} -n={\left.\;\frac{d\left\{\ln\left[-\ln\left(1-\alpha \right)\right]\right\}}{d\left[\ln\left(\beta \right)\right]}\;\right|}_{T}, \end{array}$$

with Avrami exponent *n*, reduction degree *α*, heating rate *β*, and temperature *T*. Thus, values of ln⁡[−ln⁡(1 − *α*)] were calculated at fixed temperatures and plotted as function of ln⁡(*β*). Temperature intervals were equidistant. The slopes of the resulting straight lines (Figure 14(b)) were used to determine the Avrami exponents. Plotting ln⁡[−ln⁡(1 − *α*)] as function of reciprocal temperature did not afford straight lines for sample 2.5 wt% Fe_Citrate. Therefore, JMAK kinetics were not applied to the data of this sample. Topological dimension and Avrami exponent as function of temperature and heating rate for sample 7.2 wt% Fe_Nitrate and 2.0 wt% Fe_Nitrate are depicted in Figures 15 and 16. Topological dimension and Avrami exponent for both samples were one. A topological dimension of one corresponded to linear and one-dimensional iron species in these nitrate samples. One-dimensionality was consistent with the iron species being in the pore system of SBA-15. At *n* = *m* = 1, the reduction mechanism is governed by site saturation. Thus, at the beginning of the reduction, nucleation sites either already existed or were formed immediately.

The Coats-Redfern method identified the F1, Mampel, and solid-state kinetic reaction model being suitable to describe the kinetic data. The Mampel model is consistent with the assumption of site saturation. Moreover, the Mampel model represents an exception of the Avrami-Erofeyev model with an Avrami exponent of *n* = 1. Hence, results from JMAK kinetic analysis and model-dependent Coats-Redfern method agreed well for the nitrate samples.

**Figure 17:**
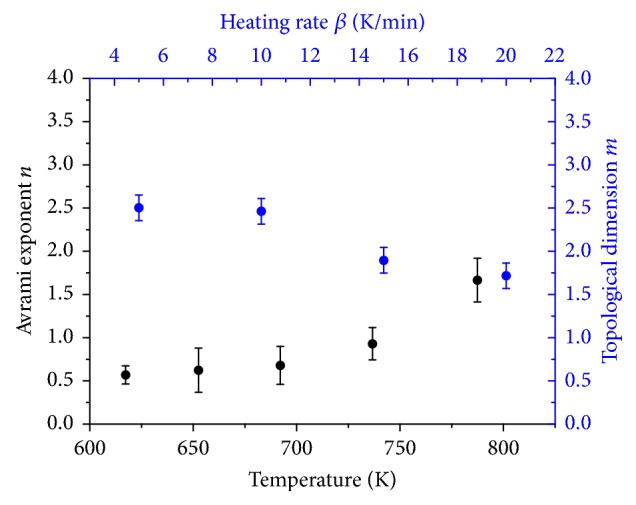
Topological dimension and Avrami exponent from JMAK kinetic analysis as function of temperature and heating rate for the mechanical mixture Fe_2_O_3_/SBA-15.

The mechanical mixture Fe_2_O_3_/SBA-15 exhibited a higher topological dimension. Topological dimension as function of the heating rate ranged between 2 and 3 (Figure 17). This increase in topological dimension correlated with the presence of Fe_2_O_3_ crystallites in this sample. The mechanical mixture exhibited Fe_2_O_3_ crystallites mixed with the support material. Model-dependent Coats-Redfern method identified the geometrical contraction model R3 being a suitable reaction model. Therefore, three-dimensional reduction was compatible with a rapid nucleation on the Fe_2_O_3_ crystallites. Thus, for the mechanical mixture Fe_2_O_3_/SBA-15, results from model-dependent Coats-Redfern analysis were confirmed by the JMAK analysis.

### 3.3. Correlation between Sample Characterization and Solid-State Kinetic Analysis

Results from sample characterization agreed well with those from solid-state kinetic analysis of the Fe_*x*_O_*y*_/SBA-15 samples. An increasing species size with increasing iron loading (DR-UV-Vis and Mössbauer spectroscopy) correlated with a decreasing apparent activation energy of reduction for the nitrate samples. Conversely, small iron species resulting from (Fe(III), NH_4_)-citrate precursor coincided with the lowest apparent activation energy for the reduction of 2.5 wt% Fe_Citrate. Sample characterization analysis methods identified the Fe(III) species as being isolated in the pore system of SBA-15 and interacting weakly with each other. Even for the higher loaded samples with more aggregated Fe_*m*_O_*n*_-nanoclusters, weakly interacting and well-dispersed Fe(III) species can be assumed. With respect to the kinetic analysis, iron species in the pores of SBA-15 react similar to isolated ions in a homogeneous solution. Accordingly, a first-order reaction model (Mampel model) was suited best to describe the similarity of the Fe_*x*_O_*y*_/SBA-15 samples and homogeneous systems. Additionally, JMAK kinetics were consistent with a one-dimensional reduction of Fe species localized in the pore system of SBA-15.

Not only for the Fe_*x*_O_*y*_/SBA-15 samples but also for the mechanical mixture Fe_2_O_3_/SBA-15, results from sample characterization agreed with those from kinetic analysis. According to JMAK analysis, the fraction of crystalline Fe_2_O_3_ in Fe_2_O_3_/SBA-15 as detected by XRD resulted in three-dimensional reduction kinetics. Hence, reduction was governed by rapid nucleation in the three-dimensional Fe_2_O_3_ crystallites. This was confirmed by the model-dependent analysis yielding a contracting volume model (R3) with rapid nucleation occurring on the surface of the Fe_2_O_3_ crystallites as suitable model for the rate-determining step in reduction.

Apparently, for both supported systems and the mechanical mixture, the results of conventional characterization and solid-state kinetic analysis corroborated each other. This showed that the concept of solid-state kinetic analysis (i.e., nonisothermal reaction conditions and model-dependent as well as model-independent methods) can be successfully applied to supported systems in addition to conventional bulk materials. Time- and temperature-dependent measurements such as TPR or TG/DTA are readily used in characterizing supported materials. Those techniques, however, yield little to no structural details of the supported species. Hence, solid-state kinetic analysis of the already available data can give additional information without additional experimental effort.

## 4. Conclusions

Iron oxides supported on SBA-15 were successfully synthesized using two different precursors (Fe(III)-nitrate and (Fe(III), NH_4_)-citrate). Independent of the precursor, an increasing size of iron species correlated with an increasing iron loading. For all Fe_*x*_O_*y*_/SBA-15 samples, a long-range ordering of iron oxidic species was excluded. Fe(III)-nitrate precursor induced larger iron oxide species. Conversely, (Fe(III), NH_4_)-citrate precursor resulted in smaller iron species accompanied by more distinct smoothing of the SBA-15 surface. Temperature-programmed reduction of the Fe_*x*_O_*y*_/SBA-15 samples revealed better reducibility of the nitrate samples compared to the citrate samples. The lowest loaded nitrate and citrate sample possessed a single-step reduction mechanism. Conversely, higher loaded Fe_*x*_O_*y*_/SBA-15 samples revealed a more complex multistep reduction mechanism.

Solid-state kinetic analysis using model-dependent and model-independent methods demonstrated their applicability to dispersed iron species on a high surface area support material. Iron species obtained from the lowest loaded citrate precursor exhibited the lowest apparent activation energy. In the series of nitrate samples, a decreasing apparent activation energy and an increasing size of the iron species correlated with an increasing iron loading. Coats-Redfern method identified the Mampel reaction model as suitable to account for the rate-determining step in reduction. Moreover, site saturation, as suggested by the Mampel reaction model, was consistent with the results of JMAK analysis (*n* = *m* = 1).

## Figures and Tables

**Table 1:** Characteristics of Fe_*x*_O_*y*_/SBA-15 samples: specific surface area, *a*_BET_ (BET method), pore volume, *V*_Pore_, and average pore radius, *r*_Pore_ (BJH method), unit cell parameter,*a* (hexagonal pore system), and difference in fractal dimension, Δ*D*_*f*_ (difference before and after deposition of iron species on SBA-15 (Table 2), modified FHH method).

Sample	*a*_*s*,BET_/m^2^/g	*V*_pore_/cm^3^/g	*r*_pore_/nm	*a*/nm	Δ*D*_*f*_
2.5 wt% Fe_Citrate	725.2 ± 0.7	1.112 ± 0.001	4.030 ± 0.004	11.15 ± 0.02	0.12 ± 0.02
6.3 wt% Fe_Citrate	650.9 ± 0.7	0.968 ± 0.001	4.030 ± 0.004	11.02 ± 0.02	0.21 ± 0.05
10.7 wt% Fe_Citrate	605.7 ± 0.6	0.898 ± 0.001	4.030 ± 0.004	10.94 ± 0.02	0.15 ± 0.01
2.0 wt% Fe_Nitrate	703.0 ± 0.7	1.096 ± 0.001	4.030 ± 0.004	11.16 ± 0.01	0.06 ± 0.01
7.2 wt% Fe_Nitrate	647.8 ± 0.6	0.971 ± 0.001	4.03 ± 0.004	11.11 ± 0.02	0.07 ± 0.01
9.3 wt% Fe_Nitrate	633.1 ± 0.6	0.939 ± 0.001	4.03 ± 0.004	11.10 ± 0.01	−0.06 ± 0.02

**Table 2:** Fractal dimension, *D*_*f*_, of all Fe_*x*_O_*y*_/SBA-15 samples and corresponding SBA-15 (modified FHH method) and difference in fractal dimension, Δ*D*_*f*_, between SBA-15 and corresponding Fe_*x*_O_*y*_/SBA-15 samples.

Sample	*D*_*f*_	*D*_*f*_ (SBA-15)	Δ*D*_*f*_
2.5 wt% Fe_Citrate	2.520 ± 0.009	2.637 ± 0.006	0.12 ± 0.02
6.3 wt% Fe_Citrate	2.350 ± 0.042	2.563 ± 0.011	0.21 ± 0.05
10.7 wt% Fe_Citrate	2.345 ± 0.039	2.497 ± 0.014	0.15 ± 0.01
2.0 wt% Fe_Nitrate	2.549 ± 0.005	2.604 ± 0.004	0.06 ± 0.01
7.2 wt% Fe_Nitrate	2.550 ± 0.004	2.617 ± 0.005	0.07 ± 0.01
9.3 wt% Fe_Nitrate	2.613 ± 0.009	2.557 ± 0.007	−0.06 ± 0.02

**Table 3:** Mössbauer parameters for 9.3 wt% Fe_Nitrate, 7.2 wt% Fe_Nitrate, 2.0 wt% Fe_Nitrate, and 10.7 wt% Fe_Citrate. Temperature, *T*, isomer shift, *δ* (referred to *α*-Fe at 298 K and not corrected for 2nd-order Doppler shift), quadrupole shift, *ε*, line widths, Γ_HWHM_, hyperfine magnetic field, *B*_hf_, fluctuation rate, *ν*_*c*_, and area. *∗* indicates values held fixed in simulation. [*a*] indicates that relaxation rate reached the dynamic limit.

Sample	*T*/K	*δ*/mm/s	*ε*/mm/s	Γ_HWHM_/mm/s	*B*_hf_/T	*ν*_*C*_/mm/s	Area/%
9.3 wt% Fe_Nitrate	300	0.320 (9)	0.173 (42)	0.29 (11)	48.3^*∗*^	[*a*]	48
		0.327 (8)	0.536 (37)	0.277 (45)	48.3^*∗*^	[*a*]	52
	14	0.401 (21)	−0.012 (20)	0.28^*∗*^	46.5 (2.7)	0.13	45
		0.465 (11)	−0.018 (11)	0.28^*∗*^	50.0 (1.2)	0.02	52
		0.401^*∗*^	0.43^*∗*^	0.37^*∗*^	48.3^*∗*^	310^*∗*^	3

7.2 wt% Fe_Nitrate	300	0.330 (5)	0.299 (15)	0.190 (16)	48.3^*∗*^	[*a*]	52
		0.307 (7)	0.508 (27)	0.233 (17)	48.3^*∗*^	[*a*]	48
	14	0.394 (35)	−0.014 (43)	0.23^*∗*^	45.4 (5)	0.3	49
		0.462 (19)	−0.034 (19)	0.23^*∗*^	49.7 (2)	0.1	39
		0.431 (72)	0.518 (59)	0.45^*∗*^	48.3^*∗*^	[*a*]	12

2.0 wt% Fe_Nitrate	300	0.336 (15)	0.346 (78)	0.273 (60)	48.3^*∗*^	[*a*]	60
		0.312 (21)	0.583 (95)	0.265 (71)	48.3^*∗*^	[*a*]	40
	14	0.421^*∗*^	0.08 (12)	0.24 (76)	43.8 (1.5)	0.7	55
		0.423 (48)	0.500 (60)	0.437 (80)	48.3^*∗*^	520	45

10.7 wt% Fe_Citrate	300	0.294 (12)	0.206 (39)	0.34 (12)	48.3^*∗*^	[*a*]	45
		0.316 (12)	0.672 (50)	0.376 (49)	48.3^*∗*^	[*a*]	55
	14	0.451^*∗*^	−0.008 (64)	0.20^*∗*^	43.5^*∗*^	5.6	34
		0.451 (10)	−0.005 (97)	0.20^*∗*^	43.5 (7)	0.5	31
		0.438 (15)	0.466 (22)	0.23^*∗*^	48.3^*∗*^	[*a*]	25
		0.416 (38)	0.814 (50)	0.23^*∗*^	48.3^*∗*^	[*a*]	10
	4	0.497 (62)	0.018 (62)	0.20^*∗*^	48.9 (5)	0.05	14
		0.424 (47)	−0.026 (45)	0.20^*∗*^	45.0 (6)	0.45	81
		0.438^*∗*^	0.47^*∗*^	0.23^*∗*^	48.3^*∗*^	[*a*]	1
		0.416^*∗*^	0.81^*∗*^	0.23^*∗*^	48.3^*∗*^	[*a*]	4

**Table 4:** Apparent activation energy of the rate-determining step in reduction of iron-containing samples in 5% H_2_ as determined by Kissinger method.

Sample	*E*_*a*_/kJ/mol
2.5 wt% Fe_Citrate	39 ± 8
2.0 wt% Fe_Nitrate	88 ± 8
7.2 wt% Fe_Nitrate	84 ± 1
9.3 wt% Fe_Nitrate	62 ± 8
Fe_2_O_3_/SBA-15	59 ± 7

**Table 5:** Apparent activation energy of reduction of sample 2.5 wt% Fe_Citrate in 5% H_2_ at various heating rates depending on the applied solid-state kinetic reaction model.

Heating rate/K/min	*E*_*a*_/kJ/mol				
	B1	R2	A2	D4	F1
5	43.8 ± 0.2	65.7 ± 0.01	32.6 ± 0.03	138.9 ± 0.1	75.0 ± 0.1
10	41.6 ± 0.3	65.7 ± 0.1	31.9 ± 0.03	140.4 ± 0.3	73.7 ± 0.1
20	54.6 ± 0.3	65.5 ± 0.1	35.7 ± 0.1	142.1 ± 0.2	77.9 ± 0.3
